# Differential effect of two dietary protein sources on time course response of muscle anabolic signaling pathways in normal and insulin dysregulated horses

**DOI:** 10.3389/fvets.2022.896220

**Published:** 2022-08-01

**Authors:** Caroline M. M. Loos, Kyle R. McLeod, Eric S. Vanzant, Sophie A. Stratton, Adam D. Bohannan, Robert J. Coleman, David A. van Doorn, Kristine L. Urschel

**Affiliations:** ^1^Department of Animal and Food Sciences, University of Kentucky, Lexington, KY, United States; ^2^Equivado Consultancy B.V., Utrecht, Netherlands

**Keywords:** protein source, mTOR, muscle, insulin dysregulation, horse

## Abstract

The objective of the study was to characterize the temporal changes of phosphorylation patterns of mTOR signaling proteins in response to two dietary protein sources in insulin dysregulated (ID, *n* = 8) and non-ID (*n* = 8) horses. Horses were individually housed and fed timothy grass hay and 2 daily concentrate meals so that protein was the first limiting nutrient and the total diet provided 120% of daily DE requirements for maintenance. On sample days, horses randomly received 0.25 g CP/kg BW of a pelleted alfalfa (AP) or commercial protein supplement (PS). Blood samples were collected before and 30, 60, 90, 120, 150, 180, 210, 240, 300, 360, 420, and 480 min post feeding and analyzed for plasma glucose, insulin and amino acid (AA) concentrations. G*luteus Medius* muscle samples were obtained before and 90, 180, and 300 min after feeding and analyzed for relative abundance of phosphorylated mTOR pathway components using western immunoblot analysis. There was no effect of protein source on postprandial glucose and insulin responses (*P* ≥ 0.14) but consumption of PS elicited a 2 times larger AUC for essential AA (EAA), greater peak concentrations of EAA and a shorter time to reach peak EAA concentrations compared to AP. Abundance of phosphorylated mTOR (*P* = 0.08) and rpS6 (*P* = 0.10) tended to be ~1.5-fold greater after consumption of PS at 90 min compared to AP. Dephosphorylation patterns differed between protein sources and was slower for AP compared to PS. ID horses had a 2 times greater (*P* = 0.009) AUC and 3 times higher postprandial peak concentrations (*P* < 0.0001) for insulin compared to non-ID horses after consumption of both treatment pellets, but EAA responses were similar between groups (*P* = 0.53). Insulin status did not affect rpS6 or mTOR phosphorylation after consumption of either protein source (*P* ≥ 0.35), but phosphorylated rpS6 abundance was twice as high in ID compared to non-ID horses (*P* = 0.007). These results suggest that the consumption of higher quality protein sources may result in greater postprandial activation of the mTOR pathway compared to equal amounts of a forage-based protein source. Moreover, ID does not impair postprandial activation of mTOR and rpS6 proteins in horses following a protein-rich meal.

## Introduction

Efficient muscle protein synthesis (MPS) is important for optimal growth, development, and performance in horses. MPS is regulated by the mechanistic target of rapamycin (mTOR) pathway involving phosphorylation of the mTOR kinase complex which subsequently activates downstream effectors, including ribosomal S6-kinase (rpS6), eventually leading to translation initiation (1, 2). It is well-known that protein ingestion and subsequent postprandial rise in essential amino acid concentrations stimulates the mTOR pathway leading to an increase in muscle protein synthesis (3, 4). Amino acids have a dual role in stimulating protein synthesis by serving both as building blocks and essential metabolic signaling molecules needed for full activation the mTOR pathway (5). In particular, leucine has been described as the most potent amino acid responsible for activation of the mTOR pathway and induction of protein translation initiation (6). Consequently, it is not surprising that MPS is affected by the source of protein as both the amino acid profile as well as protein digestibility determines the amount of specific amino acids entering circulation. Studies in humans have illustrated that ingestion of proteins that are digested more quickly, such as whey, resulted in a greater extend of mTOR pathway activation and subsequent rates of MPS compared to more slowly digested proteins, including soy, wheat, or casein (7, 8). This differential anabolic effect seems to be primarily mediated by a difference in available leucine as MPS rates were equalized between protein sources if additional leucine was supplemented to the meal (9).

It is common practice in the equine industry to add protein-dense supplements to the diet when muscle building is desired, however, to date, no studies have compared the muscle anabolic effects of different sources of protein in horses. It has been shown that the consumption of more digestible protein sources (e.g., from fortified concentrates) results in greater elevation of plasma essential amino acid concentrations and improved nitrogen balance compared to ingestion of lower digestible protein sources (e.g., from forages) (10). We have recently shown that the ingestion of a protein-rich meal activates mTOR pathway proteins in skeletal muscle of horses in a dose-dependent manner 90 min post feeding (11). However, whether mTOR phosphorylation is maximal at this timepoint in horses is currently unknown. Further characterization of the temporal changes of the phosphorylation patterns of mTOR signaling proteins will provide valuable information regarding equine muscle remodeling following protein feeding.

While it has clearly been demonstrated that the presence of amino acids is essential for protein synthesis, insulin signaling also plays a role in mTOR pathway activation (12). Several studies have illustrated that exogenous insulin administration increases phosphorylation of mTOR pathway proteins in equine muscle tissue (13, 14). Moreover, insulin resistant horses have decreased basal cell-surface GLUT-4 expression (15) and decreased glycogen-synthase kinase phosphorylation (16). However, postprandial activation of mTOR signaling components in horses with naturally occurring insulin dysregulation (ID) has not yet been investigated.

The objective of the current study was to evaluate the effect of two dietary protein sources on time course changes in muscle translation initiation in ID and non-ID horses. We hypothesized that: (1) consumption of a higher quality protein source would result in a greater intramuscular mTOR pathway activation compared to a lower quality protein source, (2) that postprandial muscle mTOR pathway activation would be maximized at 90 min followed by gradual dephosphorylation of the signaling proteins and (3) that anabolic signal transduction in response to protein meal would be diminished in ID horses.

## Materials and methods

### Animals and housing

Sixteen mature mares of mixed breed were selected from the University of Kentucky, Department of Veterinary Science research herd. Horses were identified based on their insulin status determined by an oral sugar test (OST) as previously described (17). Briefly, two blood samples were collected *via* jugular venipuncture before and 60 min after administration of 0.15 mL/kg body weight (BW) of Karo light corn syrup. Based on criteria published by the Equine Endocrinology Group (EEG), 8 insulin dysregulated (ID) and 8 non-ID horses were identified for the study (Table 1), with ID defined as plasma insulin concentrations >20 and >45 μIU/mL at baseline and 60 min post OST, respectively (18). Additionally, basal plasma adrenocorticotropic hormone (ACTH) concentrations were determined to exclude horses with pituitary pars intermedia dysfunction (PPID). Based on the recommendations by the EEG, horses with ACTH concentrations >100 pg/mL were excluded from the study (19). All horses had ACTH values <50 pg/mL with exception of one horse with levels of 80 pg/mL, which is considered “equivocal” for fall months. As this horse showed no clinical signs of PPID, the decision was made to in include this horse in the study.

**Table 1:** Phenotypic measures and insulin status of ID and non-ID groups.

**Phenotypic measure**	**Non-ID**	**ID**	***P*-values**
Insulin OST 0 min (μIU/mL)	14.4 ± 2.4	40.4 ± 2.6	<0.0001
Insulin OST 60 min (μIU/mL)	22.9 ± 4.8	96.6 ± 4.8	<0.0001
Body weight (kg)	556.1 ± 20.9	535.5 ± 20.9	0.5
Body condition score	6.1 ± 0.6	7.6 ± 0.6	0.0002
Age (years)^*^	14.0 ± 1.4	15.8 ± 1.6	0.4
Gluteal muscle fat thickness (cm)^a^	0.26 ± 0.07	0.38 ± 0.07	0.07

ID, insulin dysregulated horses; OST, oral sugar test. n = 8 non-ID and n = 8 ID horses.

* Age was not known for all horses due to limited history on horse donations (N = 7 known for non-ID, N = 4 known for ID group).

a Subcutaneous fat thickness over the gluteus medius muscle was assessed via ultrasound. P < 0.05 indicates differences between ID and non-ID group. All data are lsmeans ± SEM.

Horses were housed in individual indoor stalls (3.5 × 3.5 m) overnight and turned out into dry lot paddocks in pairs for ~6 h/day. During turnout no feed was provided but all horses had *ad libitum* access to water, salt, and mineral blocks. To accurately measure changes in muscle protein synthetic pathways, it was essential that protein was the first limiting nutrient, and horses remained in positive energy balance. Horses were individually fed and received 1.25% of BW per day (as fed) of timothy hay (Table 2) offered in a hay net. Based on the estimated digestible energy (DE) intake from the hay portion, each horse was fed 0.45% of BW of concentrate so that the total diet provided 120% of the daily DE requirements for average maintenance (20). The custom-made concentrate mixture consisted of 44% whole oats, 45% beet pulp shreds, and 11% soybean oil (Table 2). Additionally, a commercially available vitamin-mineral premix pellet (Cavalor Support, Cavalor Feeds and Supplements, Deinze, Belgium) was added to the daily meals at the manufacturer's recommended level of 100 g/day. This dietary regimen was designed to meet or exceed all nutrient needs for mature, idle horses with “average” maintenance requirements while minimizing excessive protein intake (20). Concentrate meals were fed in feed tubs at 800 and 1,600 and the daily allotment of hay was given in the afternoon. Any leftover feed present in the feed tub and hay net was collected, weighed, and recorded every morning. Horses consumed on average 4.8 g/kg BW/day of concentrate resulting in ~14.9 kcal/kg BW and 0.43 g CP/kg BW for daily DE and CP intakes (DM basis), respectively. There were no grain refusals or leftovers at any time during the study. Hay intake was on average 1.13% of BW resulting in a daily DE and CP intake of ~24.0 kcal/kg BW and 0.9 g CP/kg BW (DM basis), respectively. Total daily intake was ~114 and 105% of requirements for DE and CP, respectively. All horses were adapted to housing and diets for at least 2 weeks prior to any measurements.

**Table 2:** Nutrient composition of the daily ration on dry matter basis.

**Nutrient**	**Concentrate**	**Timothy hay**
	**% of DM**	
DE^*^ (Mcal/kg)	3.1	2.14
Crude protein	8.9	7.75
Acid detergent fiber	18.4	38.2
Neutral detergent fiber	32.4	61.7
Water-soluble carbohydrates	6.5	9.9
Ethanol-soluble carbohydrates	6.0	8.4
Starch	20.6	1.7
Non-fiber carbohydrates	49	21.2
Calcium	0.55	0.3
Phosphorus	0.25	0.3
Magnesium	0.19	0.14
Potassium	0.34	2.46
Sodium	0.011	0.006
	**PPM**	
Iron	350	151.5
Zinc	22	32.5
Copper	5	6.5
Manganese	54	32.5
Molybdenum	0.4	1.15

* DE calculated value (Pagan, 1998).

Horses were weighed on an electronic scale weekly and condition score (BCS) was assessed at the beginning and end of the study. BCS was determined on a 1–9 scale (21) by 2 independent evaluators, blinded to the experimental design. All procedures were approved by the Institutional Animal Care and Use Committee at the University of Kentucky.

### Experimental procedures

In a crossover block design, one group of 8 horses (4 ID and 4 non-ID) was studied at a time, with 2 study blocks separated by 2 months. Screening was conducted in May for block 1 horses and August for block 2 horses with study blocks running from June-Aug. and Sept.-Nov. for block 1 and 2, respectively. Horses were adapted to housing and diets for at least 2 weeks prior to the first sample collections. Approximately 14 h prior to the start of sample procedures, all feed was removed to ensure horses were in a post-absorptive state. Horses did have access to water at all times. On the morning of each sample day, horses were fitted with an intravenous jugular vein catheter and allowed to recover for about 1 h followed by collection of two basal blood samples (10 mL, −15 and 0 min pre feeding). Next, horses were lightly sedated with ~0.25–0.5 mg/kg of i.v. xylazine hydrochloride and brought into holding stocks. The biopsy sites were aseptically scrubbed and anesthetized subcutaneously with 3 mL of 12% lidocaine. A percutaneous sample of the *Gluteus Medius* muscle was collected through a single incision, using the Bergstrom needle technique as previously described (22). Subcutaneous fat thickness over the G*luteus Medius* muscle was evaluated prior to onset of the study using ultrasound imaging. Considering fat thickness only tended to differ by 0.1 cm (Table 1) between insulin groups, the standardized muscle biopsy depth (6 cm) was kept equal for all horses. After the biopsy was completed, horses were allowed to recover in their stalls. Once sedation was fully worn off, each horse was randomly assigned to receive one of two protein treatment pellets: a custom-made alfalfa pellet (Table 3, main protein source: alfalfa meal) or a commercially available protein supplement (Cavalor VitAmino; Cavalor Feeds and Supplements, Deinze, Belgium; Table 3, main protein sources: soybean meal, alfalfa meal, potato protein). Apart from the protein sources, the two treatment pellets consisted of identical ingredients and were formulated to supply a similar amount of non-structural carbohydrates. Treatment meals were fed at an amount to provide 0.25 g of CP/kg BW. We have previously shown (11) that this dose results in near-maximal mTOR pathway activation and would therefore allow for comparison of the stimulatory effect of different protein sources without overactivation of the muscle synthetic pathways. Meal consumption times were recorded for each horse and additional blood samples (10 mL) were collected at 30, 60, 90, 120, 150, 180, 210, 240, 300, 360, 420, and 480 min post feeding. Blood samples were collected into heparinized tubes, immediately put on ice and centrifuged (1,500 × g; 10 min) within 1 h after collection. Plasma was harvested and frozen (−20°C) until further analysis. Postprandial muscle samples were collected at 90, 180, and 300 min using similar procedures as described above. Muscle biopsies were performed on alternating sides of the horse for each time point and minimum 3 inches away from the previous biopsy site. Muscle samples were immediately processed, as described below, and stored at −80°C until further analysis. When horses did not consume their treatment pellets within a timely manner (<60 min), sample collection was ceased, and data were removed from final statistical analysis. After sample collection was completed, horses were fed their daily allotment of hay and evening ration of concentrate. All horses received 2 g of phenylbutazone paste (1 g/2 × daily) for 4 days after sample collections to alleviate any inflammation related to biopsy procedures. However, all biopsy sites healed quickly and normally. After 28 days of recovery, all sample procedures were repeated where each horse received the alternate treatment pellets. Muscle samples were collected from an area of the *Gluteus Medius* at least 2 inches away from any previous biopsy sites to avoid potential scar tissue. After completion of the study, all horses returned to the University of Kentucky, Department of Veterinary Science herd.

**Table 3:** Nutrient composition (dry matter basis) and macronutrient intake of each treatment pellet.

**Nutrient**	**Protein supplement pellet**		**Alfalfa pellet**	
	**Composition**	**Nutrient intake/kg BW**	**Composition**	**Nutrient intake/kg BW**
DE (Mcal/kg)*	3.2	2.08 kcal	1.8	2.55 kcal
	**% DM**	**g**	**% DM**	**g**
DM	87.8	0.66	89.7	1.46
Crude protein	38	0.25	17.2	0.25
Acid detergent fiber	13.9	0.09	33.7	0.49
Neutral detergent fiber	21.3	0.14	43.0	0.63
Water-soluble carbohydrates	9.5	0.06	5.6	0.08
Ethanol-soluble carbohydrates	8.4	0.06	4.4	0.06
Starch	7.6	0.05	3.0	0.04
Essential amino acids		**mg**		**mg**
Lysine	2.31	15.20	0.65	9.4
Leucine	3.03	19.96	1.01	14.7
Isoleucine	1.85	12.17	0.62	9.1
Valine	2.03	13.38	0.77	11.2
Threonine	1.59	10.44	0.57	8.3
Histidine	0.90	5.90	0.29	4.2
Phenylalanine	1.97	12.93	0.66	9.6
Methionine	0.59	3.86	0.21	3.1
Arginine	2.36	15.50	0.58	8.4
Tryptophan	0.49	3.25	0.16	2.3

* DE calculated value (Pagan, 1998); Main protein sources for the protein supplement pellet: soybean meal, alfalfa meal, potato protein and wheat bran. Main protein source for the alfalfa pellet: alfalfa meal.

### Sample analyses

#### Plasma glucose and insulin analyses

Plasma glucose concentrations were determined enzymatically using an automated analyzer (YSI 2700 Select Analyzer, YSI Inc., Yellow Spring, OH). Plasma insulin levels were assayed using a commercially available radioimmunoassay kit (PI-12K, Millipore Sigma, Burlington, MA) validated for horses as previously described (23). All plasma samples were run in duplicate. Average intra and inter-assay variation for glucose was 0.3 and 3.4%, respectively. Average intra and inter-assay variation for insulin was 7.6 and 7.4%, respectively.

#### Plasma amino acid analysis

Plasma free amino acid concentrations were determined by reverse-phase HPLC of phenyisothiocyanate derivatives as previously described (22). Feed amino acid analysis concentrations were determined using cation-exchange chromatography (cIEC-HPLC) coupled with post-column ninhydrin derivatization and quantitation by a commercial laboratory (Agricultural Experiment Station Chemical Laboratories, University of Missouri-Columbia, MO).

#### Western immunoblot analysis

Western immunoblot analysis of the muscle samples was performed as previously described (11). Briefly, immediately after collection, muscle samples were homogenized, centrifuged and the supernatant was removed and stored at −80°C until further analysis. Total protein content of the homogenate samples was determined using a Bradford assay (Cat. No. 97065-020, VWR International, Indianapolis, IN) and samples were diluted in Laemmli buffer until further analysis. Proteins (20 μg) of each sample were loaded on 8% (mTOR) or 12% (rpS6) sodium dodecyl sulfate (SDS) polyacrylamide gels and separated by SDS-PAGE. Gels were run at 100V for 20 min (stacking gel) after which voltage was increased to 180V and ran until the marker reached the bottom of the running gel. Proteins were then transferred to a PVDF membrane for immunoblotting. Rabbit polyclonal antibodies were used against total (1:1,000; #2972, Cell Signaling Technology, Danvers, MA) and Ser^2448^ phosphorylated (1:1,000; #2971, Cell Signaling Technology, Danvers, MA) mTOR and Ser^235/236^ and Ser^240/244^ phosphorylated (1:2,000; #2211/#2215 Cell Signaling Technology, Danvers, MA) rpS6 while a monoclonal antibody was used against total rpS6 (1:10,000; #2217, Cell Signaling Technology, Danvers, MA). Proteins were visualized and images captured using a chemiluminescent detection kit (GE Healthcare Bio-Sciences, Pittsburgh, PA) and a digital imager (Azure c600, Azure biosystems, Dublin, CA). All antibodies were previously validated in our lab for use with equine muscle tissue (11, 22). All gels were run in duplicate and data are expressed as an average of two gels. Band density of all blots were quantified by commercial densitometry software (AzureSpot, Azure Biosystems, Dublin, CA) and expressed as relative abundance of the proteins. To account for loading errors, each sample band was normalized to the total amount of protein in the gel lane of the respective sample. Additionally, each sample was normalized to a positive control that was loaded onto each gel to account for inter-gel variation.

### Statistical analyses

Net area under the response curve (AUC) was calculated as total AUC minus negative peak areas (i.e., area under baseline), using the trapezoidal method with commercially available software (GraphPad Prism, v.4). Data analysis was performed using mixed procedures in SAS 9.4 statistical software (SAS Institute, Cary, NC) with significance considered at *P* ≤ 0.05 and trends considered when 0.05 < *P* ≤ 0.10.

Screening data (Table 1) was analyzed using a one-way ANOVA to assess effects of insulin status (i.e., ID vs. non-ID, “INSstatus”) between the horse groups at the start of the study. Insulin status was modeled as the fixed effect.

Variables measured over time were analyzed using repeated measures ANOVA with appropriate variance-covariance structures chosen for each variable based on lowest AICC fit statistics, and the kr2 adjustment for degrees of freedom. Insulin status (i.e., ID vs. non-ID), treatment (i.e., protein source; “Treat”), time, and their interactions were considered fixed effects. Baseline values for each variable were included as a covariate in the model. Block and treatment sequence (i.e., which treatment pellet was received first) were considered random factors in the model with “horse^*^sample period” as the error term. Time was considered the repeated measure with horse nested in period as the subject. Differences between fixed effects for all other variables were analyzed by ANOVA with insulin status, treatment and their interactions considered as fixed effects and block, sequence, and sample period as the random effects. Data for horses that did not consume their treatment meal within 60 min were removed from the final data set. This resulted in *n* = 6 and *n* = 8 replicates for the alfalfa treatment for ID and non-ID horses, respectively. All horses consumed the protein supplement in a timely manner (*n* = 8 for both groups). For all data, where fixed effects were significant, least square means were compared using the SAS pdiff test. Relationship between blood parameters and muscle proteins were evaluated using Pearson's correlation coefficient.

Heterogeneity of variance was assessed for each response variable by evaluating the AICC criterion that resulted from alternately setting the “group” option within the repeated statement equal to each of the main effects and their interaction and without a “group” specification to test homogeneous variance models. In each case, the model with the smallest AICC value was chosen as the best-fit model, resulting in defining either “insulin status” or “treatment pellet” as the source of heterogeneity in the covariance structure. Studentized residuals for all response variables were evaluated graphically (using Q/Q plots) and with the SAS univariate procedures to ensure adherence to normality assumptions. Due to non-normal distribution of the studentized residuals, glucose data were log-transformed for analysis. Outliers were identified from studentized residuals >3 SD from the mean and were removed from the data set where appropriate. All data are presented as least square means and standard error of the mean.

## Results

### Body weight and BCS

ID horses had a higher BCS compared to non-ID horses (*P* < 0.0001), but BW was not different between groups (*P* = 0.40, Table 4). BW and BCS did not change throughout the study (*P* ≥ 0.75, Table 4).

**Table 4:** Body weight and BCS at the start and end of the study in ID and non-ID horses.

	**Start of study**		**End of study**		***P*** **-values**		
	**Non-ID**	**ID**	**Non-ID**	**ID**	**Period**	**INSstatus**	Period^*^INStatus
BW (kg)	556.1 ± 20.6	535.5 ± 20.6	550.7 ± 20.6	536.4 ± 20.6	0.91	0.40	0.90
BCS	6.1 ± 0.6	7.6 ± 0.6	5.7 ± 0.60	7.8 ± 0.6	0.75	<0.0001	0.20

BW, body weight; BCS, body condition score; ID, insulin dysregulated; INSstatus, effect of insulin status (i.e., ID vs. non-ID); Period effect compares measures at start vs. as the end of the study. n = 8 ID and 8 non-ID horses. All data are lsmeans ± SE.

### Treatment pellet intake

Sugar, starch, and CP intake was similar with both treatment meals providing 0.04 g sugar, 0.06 g starch and 0.25 g CP/kg BW/meal (Table 3). To provide similar amounts of CP, the average meal size was 0.9 and 0.4 g per kg of BW for alfalfa and protein supplement pellets, respectively. Consequently, meal consumption time for alfalfa pellets was greater (17 ± 1.27 min, *P* < 0.0001) than that for the protein supplement (4.62 ± 1.15 min). There was a treatment by insulin status interaction on meal consumption times (*P* = 0.01, Table 5). While all horses took longer to eat the alfalfa pellets compared to the protein supplement pellets (*P* ≤ 0.003), non-ID horses took ~2× longer (21.7 ± 1.27 min; *P* = 0.0012) to eat the alfalfa pellets compared to ID horses (12.37 ± 1.27 min). There was no difference between the groups in meal consumption time for the protein supplement (*P* = 0.80).

**Table 5:** Effects of protein source and insulin status on plasma insulin, glucose, EAA, leucine and non-EAA concentrations.

	**Alfalfa pellets**		**Protein supplement**		***P*** **-values**		
	**Non-ID**	**ID**	**Non-ID**	**ID**	**Treat**	**INSstatus**	Treat^*^INSstatus
Meal consumption time (min)	21.7 ± 1.73	12.4 ± 1.87	4.9 ± 1.62	4.3 ± 1.62	<0.0001	0.008	0.02
**Insulin**							
Peak conc. (μIU/mL)	27.1 ± 11.24	95.0 ± 12.98	38.3 ± 11.24	119.3 ± 11.24	0.14	<0.0001	0.58
Time to peak (min)	119.4 ± 20.14	65.0 ± 20.95	63.7 ± 20.14	65.6 ± 19.26	0.08	0.10	0.08
Net AUC	2808.1 ± 1276.87	5578.5 ± 1474.4	3198.0 ± 1276.87	8065.4 ± 1365.03	0.30	0.009	0.44
**Glucose**							
Peak conc. (mmol/L)	6.8 ± 0.51	7.2 ± 0.55	6.7 ± 0.51	7.5 ± 0.51	0.68	0.13	0.61
Time to peak (min)	118.1 ± 29.88	132.5 ± 34.41	136.9 ± 29.88	116.0 ± 30.84	0.97	0.92	0.57
Net AUC	293.8 ± 122.1	183.0 ± 131.91	322.7 ± 122.1	320.5 ± 122.1	0.36	0.54	0.55
**Essential AA**							
Peak conc. (μmol/L)	951.8 ± 58.82	991.8 ± 67.91	1158.3 ± 58.82	1146.4 ± 58.82	0.007	0.82	0.70
Time to peak (min)	221.2 ± 37.80	265.0 ± 43.12	120.0 ± 37.81	150.0 ± 37.81	0.008	0.33	0.85
Net AUC	39,507 ± 15,735	43,861 ± 16,593	68,943 ± 15,735	76,659 ± 15,735	0.003	0.53	0.86
**Leucine**							
Peak conc. (μmol/L)	116.5 ± 10.20	136.6 ± 11.55	145.2 ± 10.20	159.0 ± 10.41	0.01	0.10	0.75
Time to Peak (min)	198.7 ± 35.43	187.5 ± 40.87	131.2 ± 35.43	112.5 ± 35.43	0.06	0.68	0.92
Net AUC	5765.2 ± 3182.71	2114.8 ± 3320.98	7128.2 ± 3182.71	8291.1 ± 3182.71	0.04	0.47	0.17
**Non-essential AA**							
Peak conc. (μmol/L)	2741.6 ± 375.25	3071.9 ± 391.71	2897.4 ± 375.25	3319.6 ± 375.25	0.33	0.07	0.83
Time to Peak (min)	221.2 ± 46.07	275.0 ± 53.20	165.0 ± 46.07	168.7 ± 46.07	0.10	0.55	0.61
Net AUC	82573 ± 38462	71730 ± 41968	138623 ± 38462	169530 ± 38462	0.02	0.74	0.50

AA, amino acids; INSstatus, effect of insulin status; Treat, effect of protein source; AUC, area under the curve; conc., concentration; ID, insulin dysregulated. Essential AA: sum of all essential amino acids (i.e., histidine, threonine, valine, methionine, isoleucine, leucine, phenylalanine, tryptophan, lysine); non-essential AA: sum of all non-essential amino acids (i.e., aspartate, glutamate, serine, asparagine, glycine, glutamine, citrulline, alanine, arginine, proline, tyrosine, ornithine). n = 8 ID and 8 non-ID horses. All data are lsmeans ± SE.

### Plasma glucose and insulin concentrations

There was a trend (*P* = 0.08, Table 5) for a treatment by insulin status interaction on time to reach peak postprandial insulin concentrations. Concomitant with longer consumption times, non-ID horses took almost twice as long (*P* = 0.02) to reach peak insulin concentrations after alfalfa pellet consumption compared to ID horses. However, time to reach peak insulin concentrations was not different between groups for the protein supplement (*P* = 0.93). There were no other significant interactions between protein source and insulin status for any glucose or insulin measures (*P* ≥ 0.44).

#### Effect of protein source

Dietary protein source did not affect postprandial peak glucose or insulin concentrations, time to peak glucose concentrations or net AUC for glucose or insulin (*P* ≥ 0.14, Table 5). Additionally, there were no differences in plasma glucose or insulin concentrations between protein sources at the times of biopsy (*P* ≥ 0.22, data not shown).

#### Effect of insulin status

As expected, there was an effect of insulin status on all insulin response variables, where ID horses had ~3 times higher postprandial peak insulin concentrations (*P* < 0.0001) and 2 times greater AUC (*P* = 0.009) for insulin compared to non-ID horses (Table 5). There was no effect of insulin status on peak concentrations, time to peak or net AUC for plasma glucose (*P* ≥ 0.13). ID horses had an overall greater (*P* < 0.0001) insulin response at all times of biopsy, with average plasma insulin concentrations of 50.6 ± 5.68 and 16.69 ± 2.65 μIU/mL for ID and non-ID horses, respectively. There was no effect of insulin status on plasma glucose concentrations at times of biopsy (*P* = 0.28).

#### Effect of time

**Figure 1:**
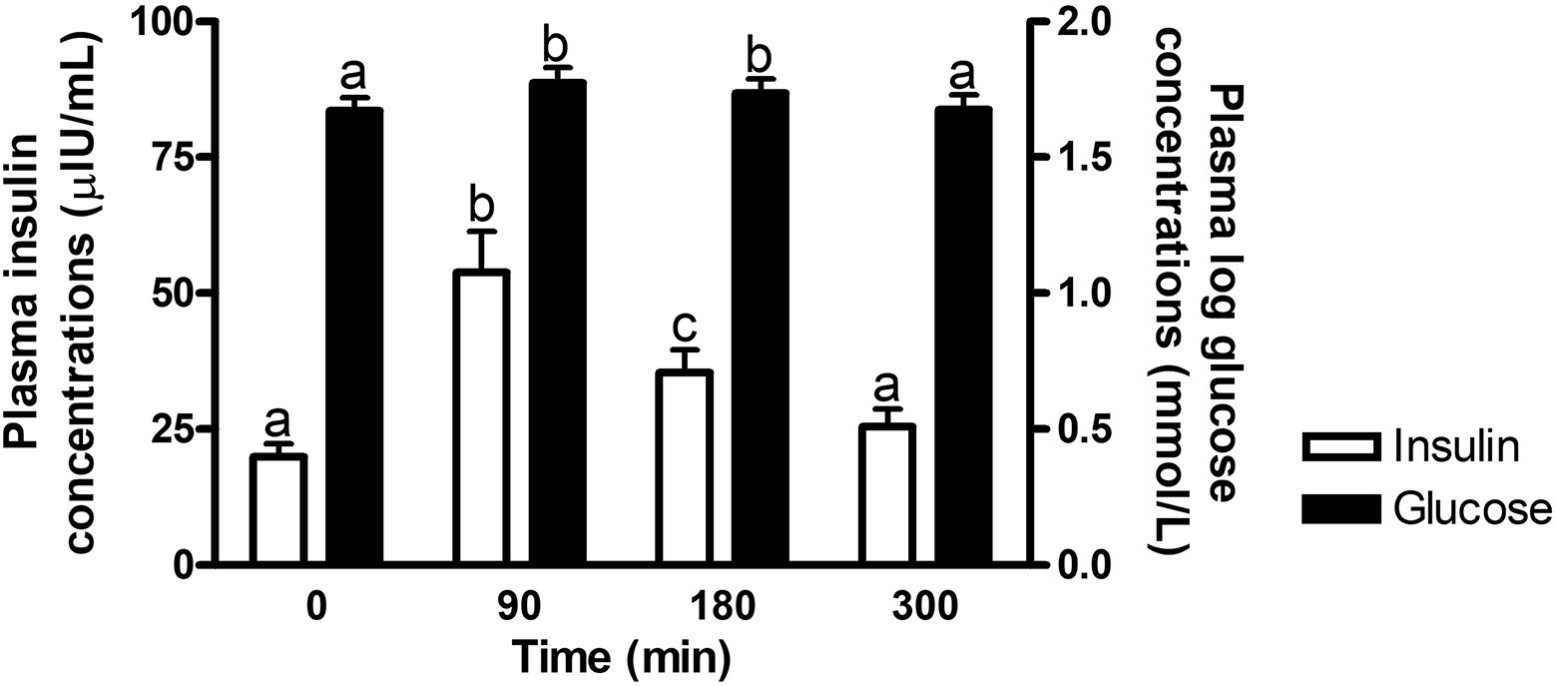
Plasma glucose and insulin concentrations at times of biopsy. Effect of time on plasma glucose (black bars) and insulin (white bars) concentrations at 0, 90, 180, and 300 min post consumption of the treatment pellets in all horses. ^a,b^Different letters indicate differences between timepoints for plasma glucose and insulin concentrations (Effect of time, *P* ≤ 0.0002). Glucose concentrations were log transformed for analysis. Data presented as least square means ± standard error of the mean.

There was an overall effect of time (*P* ≤ 0.0002) on postprandial glucose and insulin concentrations at times of biopsy (Figure 1). Glucose and insulin concentrations increased (*P* ≤ 0.009) over baseline concentrations at 90 and 180 min post feeding, but returned to basal levels by 300 min (*P* ≥ 0.37). No other interactions with time were significant (*P* ≥ 0.14).

### Plasma amino acid concentrations

There were no significant interactions (*P* ≥ 0.23) between protein source and insulin status for any of the amino acid measures.

#### Effect of protein source

**Figure 2:**
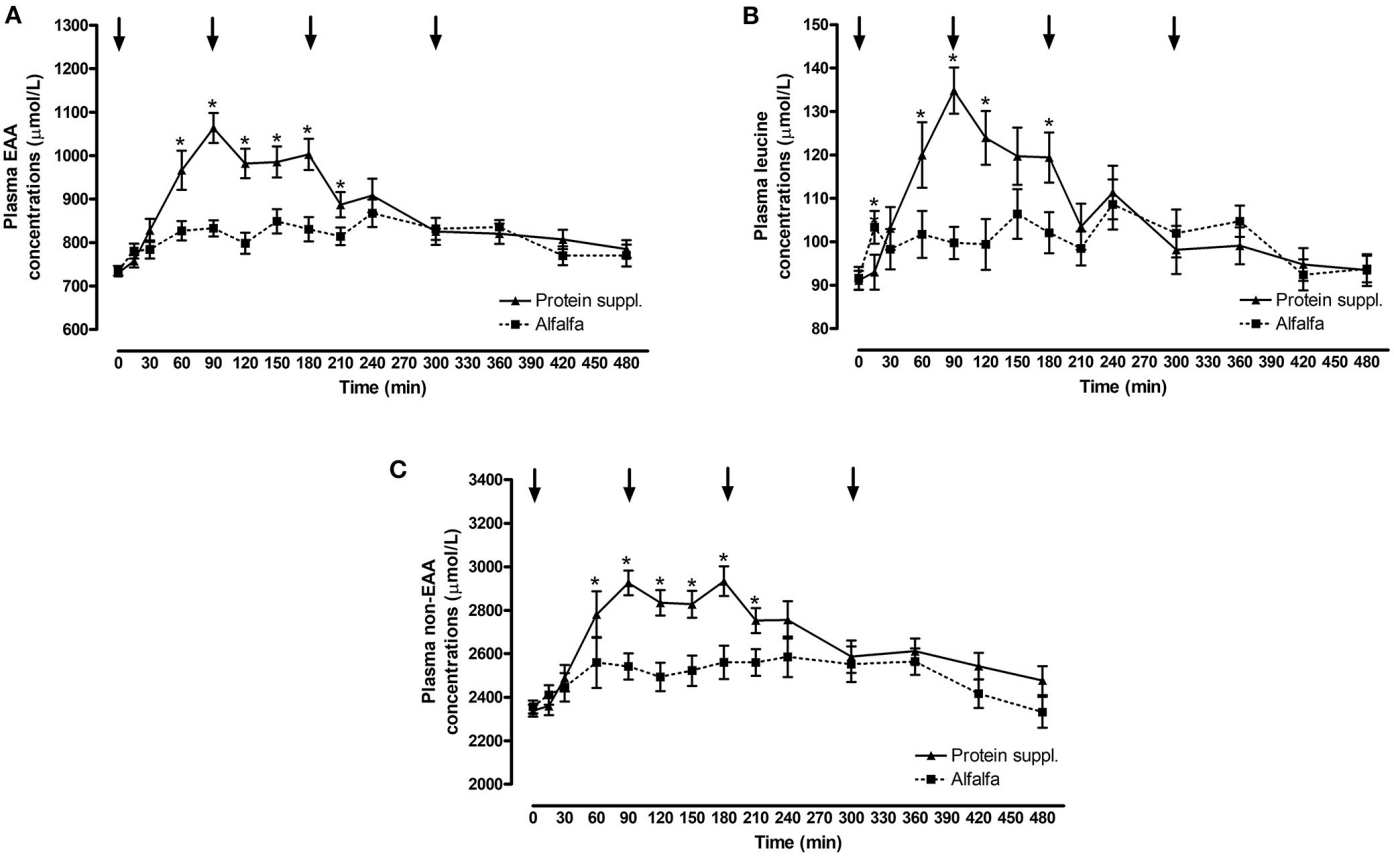
Plasma essential amino acids, leucine and non-essential amino acid responses over time after consumption of a protein supplement or alfalfa pellets. Effect of treatment by time interaction on plasma essential amino acid (EAA, **A**), leucine **(B)** and non-essential amino acids (non-EAA, **C**) concentrations post consumption of the protein supplement (solid line, *n* = 16) and alfalfa pellets (dashed line, *n* = 14) in all horses. Black arrows indicate timepoints of muscle biopsy. *Indicates differences between treatments within a timepoint. Effect of treatment by time interaction, EAA: *P* < 0.0001; leucine: *P* < 0.0001; non-EAA: *P* = 0.007. Data presented as least square means ± standard error of the mean.

There was a significant effect of treatment by time interaction (*P* ≤ 0.007) for plasma essential amino acid (EAA), leucine and non-EAA concentrations (Figure 2 and Supplementary Table 1). Plasma EAA and non-EAA concentrations increased after consumption of both treatment pellets and were elevated over basal concentrations at all biopsy timepoints (*P* ≤ 0.03) for the alfalfa and the protein supplement. EAA and non-EAA concentrations returned to basal values 420 min after consumption of the alfalfa pellet (*P* ≥ 0.17) but EAA (*P* = 0.02) and non-EAA (*P* = 0.06) were still elevated over basal levels 480 min post consumption of the protein supplement. Plasma leucine concentrations increased after consumption of both treatment pellets and were elevated (*P* ≤ 0.05) above baseline at the 90 and 180 min muscle sample collection timepoints for the alfalfa and the protein supplement. Leucine concentrations at the 300 min biopsy timepoint tended to be higher than baseline for the alfalfa treatment (*P* = 0.08) but were returned to basal levels for the protein supplement (*P* = 0.21).

Plasma EAA, leucine and non-EAA were not different between the protein sources at baseline (*P* ≥ 0.72) but consumption of the protein supplement resulted in higher plasma EAA concentrations at 60, 90, 120, 150, 180, and 210 min (*P* ≤ 0.05, Figure 2A), higher leucine concentrations at 15, 60, 90, 120, and 180 min (*P* ≤ 0.05, Figure 2B), and higher non-EAA concentrations at 90, 120, 150, 180, and 210 min (*P* ≤ 0.03, Figure 2C) post feeding compared to the alfalfa pellets. At time of biopsy specifically, plasma EAA and leucine concentrations were ~ 1.3-fold higher after consumption of the protein supplement at 90 (*P* ≤ 0.0004) and 180 min (*P* = 0.01), compared to alfalfa pellets. At 300 min there were no differences (*P* ≥ 0.61) in concentrations of EAA and leucine between the 2 protein sources.

Consumption of the protein supplement elicited greater peak (*P* ≤ 0.01, Table 5) plasma EAA and leucine concentrations compared to the alfalfa pellets. Time to reach peak plasma EAA, leucine and non-EAA amino acid concentrations was shorter (*P* ≤ 0.10, Table 5) after protein supplement consumption compared to alfalfa pellets. Similarly, net AUC for EAA, leucine and non-EAA responses was ~2-fold larger (*P* ≤ 0.04, Table 5) after eating the protein supplement compared to the alfalfa pellets. Despite greater AUC for the protein supplement, there was no difference (*P* = 0.33) in peak non-EAA concentrations between protein sources.

#### Effect of insulin status

There was a tendency for an effect of insulin status on plasma amino acid concentrations where ID horses tended to have slightly higher postprandial peak plasma leucine (*P* = 0.10, Table 5) and non-EAA concentrations (*P* = 0.07, Table 5), compared to non-ID horses. There was a trend (*P* = 0.10) for an insulin status by time interaction for plasma leucine concentrations at time of biopsy (data not shown), where plasma leucine concentrations rose above baseline levels (*P* ≤ 0.01) at 90 and 180 min in both groups. By 300 min plasma leucine concentrations were not different from baseline in the non-ID horses (*P* = 0.20) but tended to remain higher than baseline concentrations at 300 min in the ID horses (*P* = 0.06). This resulted in a tendency (*P* = 0.10) for ID (107.9 ± 7.1 μmol/L) horses to have higher leucine concentrations at 300 min compared to non-ID horses (92.5 ± 6.8 μmol/L). There was no difference in leucine concentrations between the insulin groups at any of the other time points (*P* ≥ 0.23). There was no effect of insulin status on any other AA measurements (*P* ≥ 0.33).

### Muscle MTOR pathway activation

There was no significant interaction (*P* ≥ 0.35) between protein source and insulin status for any of the western blot measures. There were no significant 3-way interactions between protein source, insulin status and time or between insulin status and time (*P* ≥ 0.3) for any of the western blot measures.

#### Effect of protein source

There was a trend (*P* = 0.08) for an overall effect of protein source on activation (i.e., abundance of phosphorylated protein) of rpS6 protein, where consumption of the protein supplement (0.62 ± 0.15) resulted in 1.3-fold greater abundance of phosphorylated rpS6 (rpS6-P; 0.46 ± 0.14) compared to alfalfa pellets (Supplementary Table 1).

**Figure 3:**
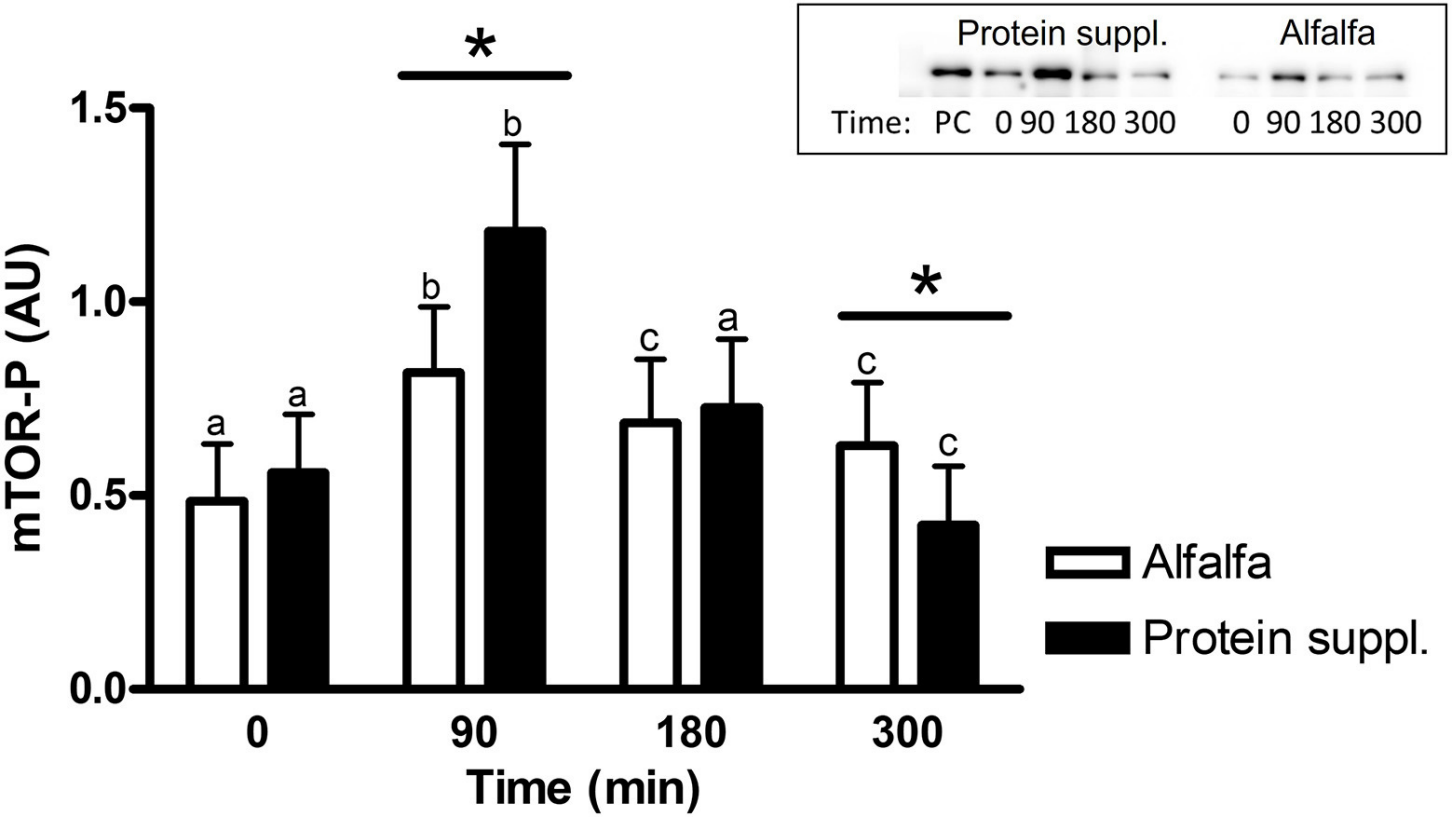
Relative abundance of phosphorylated mTOR protein in gluteal muscle samples post consumption of alfalfa pellets vs. a protein supplement. Effect of treatment by time interaction on gluteal muscle abundance of phosphorylated mTOR protein at 0, 90, 180, and 300 min post consumption of the protein supplement (black bars, *n* = 16) and alfalfa pellets (white bars, *n* = 14) in all horses. Results from a representative blot are shown as an inset. Each lane on the blot represents a time point from the same horse. PC: positive control. ^a,b,c^Different letters indicate differences between timepoints within each protein source. *Indicates differences between protein sources within a timepoint. Effect of treatment by time interaction, *P* = 0.03. Values for abundance of phosphorylated mTOR were normalized to total protein. AU; arbitrary units. Data presented as least square means ± standard error of the mean.

**Figure 4:**
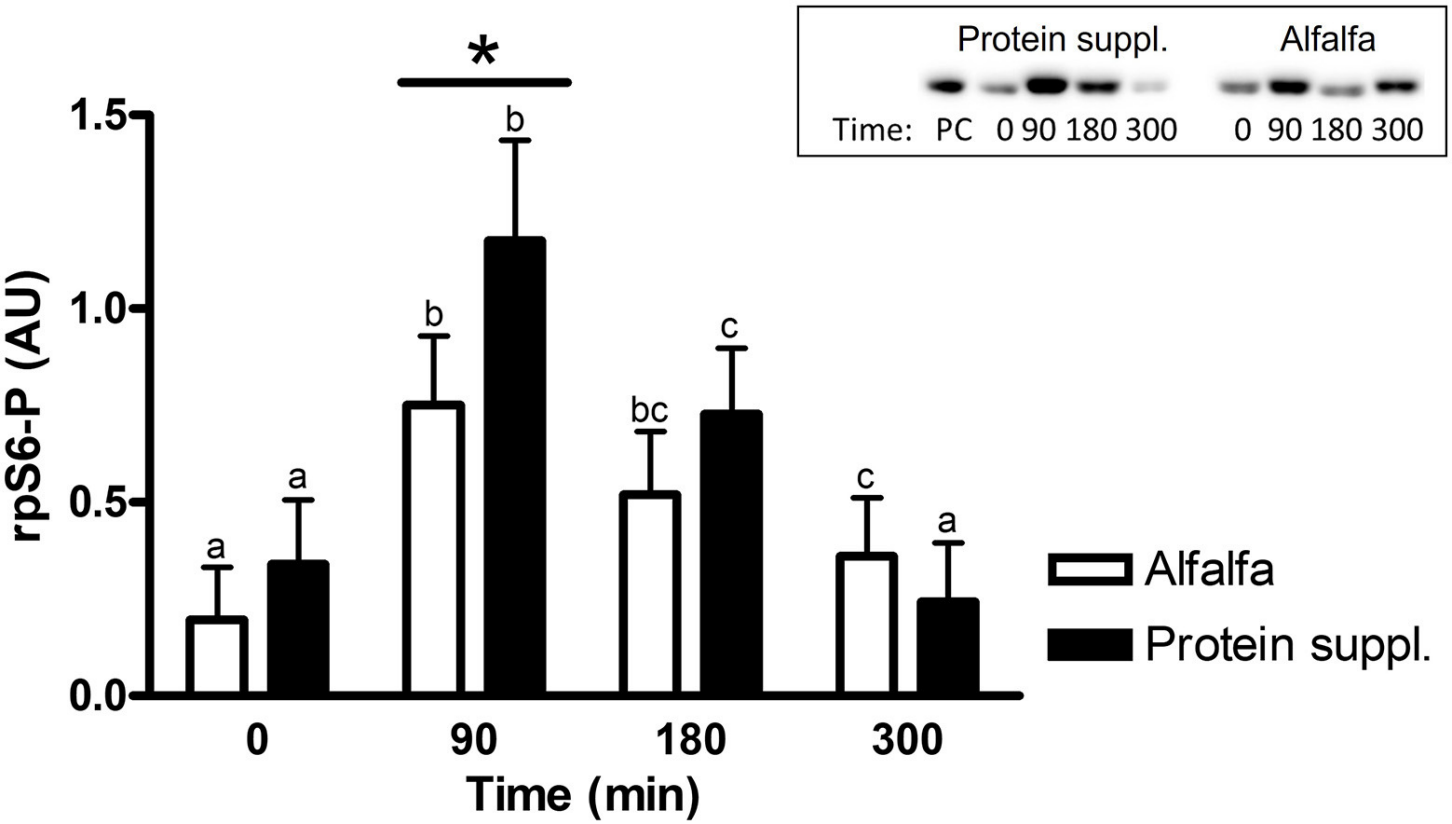
Relative abundance of phosphorylated rpS6 protein in gluteal muscle samples post consumption of alfalfa pellets vs. a protein supplement. Effect of treatment by time interaction on gluteal muscle abundance of phosphorylated rpS6 protein at 0, 90, 180, and 300 min post consumption of the protein supplement (black bars, *n* = 16) and alfalfa pellets (white bars, *n* = 14) in all horses. Results from a representative blot are shown as an inset. Each lane on the blot represents a time point from the same horse. PC: positive control. ^a,b,c^Different letters indicate differences between timepoints within each protein source. *Indicates differences between protein sources within a timepoint. Effect of treatment by time interaction, *P* = 0.10. Values for abundance of phosphorylated rpS6 were normalized to total protein. AU, arbitrary units. Data presented as least square means ± standard error of the mean.

There was a treatment by time interaction on activation of phosphorylated mTOR (mTOR-P; *P* = 0.03) and rpS6 (*P* = 0.10) proteins in the gluteal muscle samples. Abundance of mTOR-P and rpS6-P proteins increased with consumption of both treatment pellets (Figures 3, 4 and Supplementary Table 1). Levels of mTOR-P and rpS6-P were increased over baseline values at all biopsy time points after consumption of alfalfa pellets (*P* ≤ 0.09). Consumption of the protein supplement significantly increased mTOR-P at 90 min (*P* = 0.003, Figure 3) but levels returned to basal values by 180 min (*P* = 0.16) and were slightly lower than baseline values at 300 min (*P* = 0.07). Postprandial abundance of rpS6-P was increased over basal levels at 90 (*P* = 0.002, Figure 4) and 180 min (*P* = 0.01) but returned to baseline by 300 min (*P* = 0.43) with consumption of the protein supplement. There was no difference in mTOR-P or rpS6-P abundance between treatments at baseline (*P* ≥ 0.19), but mTOR (*P* = 0.08) and rpS6 (*P* = 0.10) activation tended to be ~1.5-fold greater after consumption of the protein supplement at 90 min compared to eating alfalfa pellets. By 180 min there were no differences in mTOR-P (*P* = 0.79) or rpS6-P (*P* = 0.16) levels between protein sources. At 300 min there was no difference between treatments for rpS6-P (*P* = 0.29) abundance, but mTOR-P (*P* = 0.07) tended to drop to slightly lower levels after consumption of protein supplement compared to alfalfa pellets.

#### Effect of insulin status

**Figure 5:**
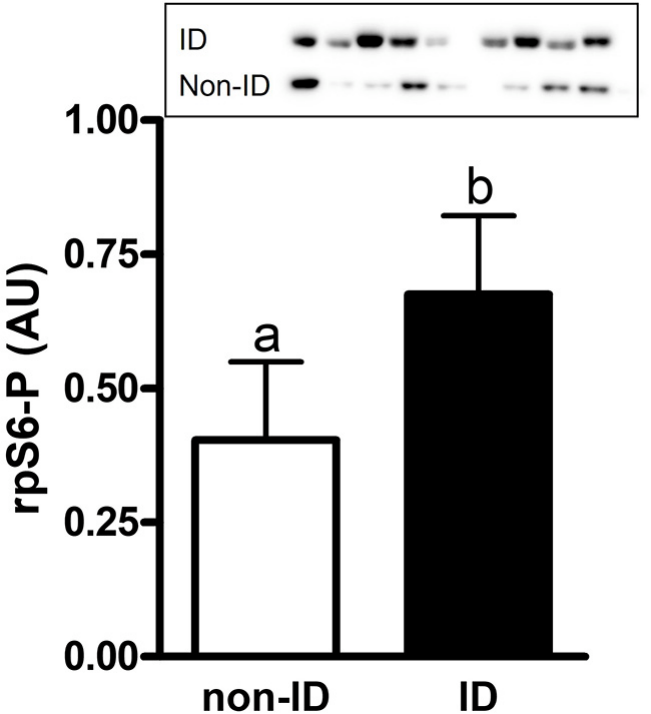
Relative abundance of phosphorylated rpS6 protein in gluteal muscle samples of ID vs. non-ID horses. Effect of insulin status on overall gluteal muscle abundance of phosphorylated rpS6 protein post consumption of the treatment pellets in ID (black bars, *n* = 8) and non-ID (white bars, *n* = 8) horses. Results from two representative blots are shown as an inset. Each lane on the blot represents a time point from the same ID and non-ID horse. ^a,b^Different letters indicate differences between insulin groups. Effect of insulin status, *P* = 0.007. Values for abundance of phosphorylated rpS6 were normalized to total protein. AU; arbitrary units. Data presented as least square means ± standard error of the mean.

There was an effect of insulin status on the abundance of rpS6-P where ID horses had ~1.7-fold higher rpS6-P levels compared to non-ID horses (*P* = 0.007, Figure 5 and Supplementary Table 1). There was no effect of insulin status on rpS6-T, mTOR-P or mTOR-T (*P* ≥ 0.18, data not shown).

Regardless of protein source or insulin status, there was greater correlation between phosphorylated protein abundance and plasma leucine concentrations (mTOR: *P* = 0.008, *r* = 0.49; rpS6: *P* = 0.05, *r* = 0.38) compared to plasma insulin concentrations (mTOR: *P* = 0.64, *r* = 0.09, rpS6: *P* = 0.09, *r* = 0.32) at 90 min.

#### Effect of time

There was an effect of time on total rpS6 (*P* = 0.008, data not shown) abundance and a trend for treatment by time interaction on abundance of total mTOR (*P* = 0.10, data not shown). Total rpS6 protein abundance was slightly elevated at 90 (*P* = 0.04) and 180 min (*P* = 0.08) before returning to baseline. The trend for treatment by time interaction showed no difference between protein sources (*P* ≥ 0.18) for total mTOR protein abundance at any time point and no differences between timepoints within the alfalfa treatment (*P* ≥ 0.20). However, total mTOR protein abundance was slightly decreased at 180 (*P* = 0.02) and 300 min (*P* = 0.01) compared to baseline for protein supplement.

## Discussion

The muscle protein synthetic response to protein ingestion is impacted by several factors, including protein digestibility (24), amino acid (AA) absorption kinetics (25), delivery to and uptake of circulating AA by the muscle (26), and efficiency of signal transduction through intramuscular regulatory pathways (1). Consequently, protein quality and quantity have a significant impact not only on the availability of the building blocks (i.e., AA) needed for *de novo* protein synthesis, but also on the magnitude and duration of postprandial stimulation of muscle protein synthesis (MPS). The main regulator of MPS is the mTOR pathway, which is activated by amino acids and insulin following a protein-rich meal (12). Activation of the mTOR protein kinase leads to phosphorylation/activation of several key downstream effectors which regulate protein translation initiation and elongation (4). It has been extensively shown in humans and rodents that degree of mTOR activation and subsequent rate of MPS are sensitive to level of protein intake and type of protein source consumed (8, 27, 28). Ingestion of increasing amounts of protein typically results in dose-dependent stimulation of muscle protein synthetic pathways (29). We have recently illustrated this concept in horses, with maximal phosphorylation of mTOR and its downstream effectors reached at ~0.25 g crude protein (CP)/kg BW. Secondly, the digestibility and amino acid profile (i.e., protein “quality”) of the protein source has been shown to differentially affect muscle anabolic pathways in other species (30). More rapidly digested protein will generally provide a stronger stimulus for MPS, attributed to a faster, more substantial increase in circulating essential AA (EAA) (25). The objective of the current study was to test this concept in horses, comparing two protein sources of different quality (i.e., AA profile and digestibility) commonly used in equine diets. We therefore chose a commercially available high protein pelleted feed supplement (soy, alfalfa, potato protein mix) and a custom-made alfalfa pellet. Both sources were fed at an equal level of CP previously established to result in near-maximal degree of mTOR protein phosphorylation in horses (11). It should be noted that for the two treatment pellets to supply equal amounts of CP alfalfa pellets were fed in a larger quantity. Consequently, consumption of the alfalfa treatment meal took longer than the protein supplement.

Legumes, such as alfalfa typically contain a higher amount of soluble protein compared to grasses, making them an overall higher quality forage source for horses with elevated nutritional needs. However, prececal alfalfa protein digestibility in horses is still reported to be ~2× lower than that of higher quality protein sources such as soybean meal (31, 32). Although digestibility was not measured in this study, it was assumed that AA bioavailability of the commercial protein supplement (i.e., soybean meal, alfalfa meal, potato protein) would be greater than the forage-associated protein (i.e., alfalfa). In line with this hypothesis, there was a rise in both plasma EAA and non-EAA concentrations following ingestion of both dietary protein sources, but the relative increase in plasma AA was greater following ingestion of the protein supplement compared to the alfalfa pellets. These results are similar to a previous study were plasma concentrations of several EAA were greater when horses received a concentrate meal (main protein source: soybean meal) compared to an isonitrogenous amount of protein coming from long stem grass hay (10). Likewise, we observed higher peak plasma EAA concentrations which was reached ~2 h faster when horses consumed the protein supplement compared to the alfalfa pellets, suggesting faster protein digestion, absorption, and/or release of EAA into circulation. Of all EAA, leucine is known to potently activate the mTOR pathway and protein translation initiation in human and animal species, including horses (33–35). The postprandial plasma leucine response followed a similar trend to that of the EAA, with a greater peak and shorter time to reach peak following consumption of the protein supplement. Interestingly, plasma EAA concentrations did not return to baseline values until 7–8 h post feeding. Similar results were seen in previous studies in horses receiving a protein-rich meal (10, 23), although the amount of CP/kg BW was significantly lower in this study. A similar time course for release of AA into circulation over a 5–7.5 h period post feeding has been described for high quality protein sources such as whey, casein and milk in humans (25).

There was a modest elevation in plasma glucose concentrations post feeding, but this was not different between protein sources, indicating that the formulation of both pellets to deliver equal amounts of sugar and starch was successful. In synergy with an increase in plasma glucose, the influx of AA lead to a significant increase in plasma insulin concentrations after consumption of both protein treatments. The insulinotropic effect of AA has been well-documented (36), and it was noticeable that consumption of the protein supplement resulted in numerically higher and faster peak plasma insulin levels compared to consumption of alfalfa pellets. With non-structural carbohydrate (NSC) levels kept consistent between protein sources, a greater increase in plasma AA concentrations was expected to have a more potent stimulatory effect on pancreatic insulin release. This has been documented in human studies, where consumption of “faster” digested proteins or free amino acids resulted in greater elevation of blood insulin concentrations compared to “slower” digested proteins (7, 37). To the best of our knowledge no studies exist to date comparing insulin responses to different isolated protein sources in horses, however, higher insulin responses were observed after nasogastric intubation with a glucose + leucine bolus vs. a glucose + whey protein bolus in thoroughbred horses (38). Additionally, horses consuming forages higher in CP had a more elevated postprandial insulin response compared to consumption of forages with lower CP (39, 40). These data suggest that protein quality certainly has the potential to impact the postprandial insulin responses and subsequent anabolic response to a protein-rich meal in horses.

As expected, the rapid and more pronounced hyperaminoacidemia following consumption of the higher quality protein supplement provided a stronger anabolic signal to the muscle, resulting in a trend toward greater abundance of phosphorylated mTOR and rpS6 proteins 90 min post feeding compared to alfalfa pellets. This is in line with studies in other species, where “fast” digestible proteins have a greater stimulatory effect on mTOR pathway protein activation and subsequent MPS compared to equal amounts of “slow” digestible proteins (7, 8, 41, 42). It should be noted that most of these studies compared animal to plant-based protein sources and there is still a paucity of data regarding the impact of different plant-based protein sources on mTOR and MPS, in particular as it pertains to herbivore diets. The observed differential effect of the protein sources on mTOR pathway stimulation is likely attributed to differences in leucine content and postprandial leucinemia, a concept that has been extensively shown in humans and other animal species (43–46). Analysis of the treatment pellets confirmed that the protein supplement had an overall superior EAA profile (Table 3), providing 61 and 80 mg of lysine and leucine per gram of CP (DM), respectively, compared to 38 and 60 mg for the alfalfa pellets. The higher leucine content of the protein supplement led to greater peak in plasma leucine concentrations and thus likely contributed to greater postprandial mTOR pathway activation. Although the inherent insulinotropic nature of certain AA prevents distinction between the stimulatory effect of insulin and AA, there was no statistical difference in the postprandial plasma insulin response between protein sources. Conversely, plasma EAA and leucine concentrations were significantly higher after consumption of the protein supplement compared to the alfalfa pellets. Previous work has suggested that the role of insulin in the activation of the mTOR pathway is suggested to be primarily permissive, with leucine proven to be the main driver of EAA-induced stimulation of mTOR and subsequent MPS (35, 47). In further support of this, peak mTOR and rpS6 activation in the current study were more correlated with plasma EAA and leucine concentrations with little to no relationship to plasma insulin concentrations. This suggests that the differential effect of protein source on mTOR pathway activation in the current study was primarily attributed to differences in EAA profile and absorption kinetics. While we did not measure rates of MPS, it is widely accepted that an increase in MPS coincides with phosphorylation of mTOR and its downstream effectors (4, 48, 49) and that greater rates of MPS is concomitant with higher degree of phosphorylation of mTOR signaling proteins (8, 43, 46). It is therefore reasonable to assume that greater mTOR pathway activation following protein supplement ingestion would be reflected by higher rates of MPS in these horses.

Differences in mTOR pathway activation between protein sources was most evident 90 min post feeding. To the best of our knowledge, this study is the first to evaluate the postprandial temporal changes in phosphorylation patterns of muscle protein synthetic pathways in horses. Regardless of protein source, there was a postprandial increase in the phosphorylation of mTOR pathway components. Peak activation was reached approximately 90 min post feeding, at which point protein phosphorylation was increased 2.0 and 4.8-fold from baseline for mTOR and rpS6, respectively. This timeline is in accordance with reports in humans, pigs, and rodents where peak activation of muscle signaling proteins was observed around 30–90 min following a protein meal (8, 48, 50). Interestingly, in addition to a greater extend of activation, phosphorylation levels of mTOR and rpS6 declined more rapidly after consumption of the protein supplement, by 180 and 300 min, respectively, while they remained higher than baseline levels up until 300 min after ingestion of the alfalfa pellets. It could be speculated that a more sustained activation of the mTOR pathway after eating alfalfa pellets could be due to longer consumption times and slower digestion which might have resulted in more gradual release of EAA into circulation. Indeed, plasma EAA concentrations only reached peak 4 h after consumption of alfalfa pellet. Prolonged activation of mTOR is typically associated with continued elevation of plasma leucine concentrations (3, 8, 48). However, while plasma leucine concentrations were higher than basal levels at 180 min, they returned to basal levels by 300 min for both treatments. Furthermore, leucine concentrations were higher for the protein pellet compared to alfalfa pellet at 180 min. Without a difference in insulin concentrations at these time points, this suggests that prolonged phosphorylation of mTOR signaling proteins with alfalfa consumption cannot solely be attributed to circulating plasma leucine or insulin concentrations. A more likely explanation would be treatment differences in cellular feedback mechanisms. In contrast to the well-defined signaling cascade leading to an increase in mTOR pathway activation and MPS, the cellular processes involved in protein dephosphorylation and decline in MPS are less well-understood. Through negative feedback loops, protein phosphatases play an important role in balancing kinase-driven phosphorylation events, thereby regulating protein translation and cell growth (51, 52). Similar to kinase activity, it is likely such phosphatases are sensitive to extend of the anabolic input. For example, excess amino acid influx has shown to cause a strong increase in the S6K1-mediated negative feedback loop, thereby regulating cellular signal transduction (53). It is therefore reasonable to assume that hyperactivation of mTOR and rpS6 90 min following consumption of the protein supplement could have resulted in a greater negative feedback response *via* protein phosphatases and faster protein dephosphorylation compared to alfalfa pellets. Whether more sustained submaximal mTOR activation has physiological relevance with regards to MPS is debatable and seems to be dependent on protein source. By use of intrinsically labeled protein, a recent study in humans elegantly illustrated that sustained aminoacidemia following consumption of slower digesting, mixed protein meal stimulated mTOR phosphorylation and MPS for up to 5 h (49). However, this study made no comparison with a faster digested protein meal. In fact, when the effect of gradual and prolonged aminoacidemia is compared to rapid and pronounced hyperaminoacidemia, it is evident that the later results in greater rpS6 phosphorylation and higher overall rates of MPS (43). These data are line with observations in the current study and suggests that the pattern of postprandial aminoacidemia, rather than net AA exposure or total protein consumption is the main determinant for the degree of muscle protein accretion. Further research is needed to determine the long-term impact of different protein sources on muscle mass development in horses.

As expected, horses with insulin dysregulation (ID) had greater postprandial plasma insulin responses to both protein sources, compared to non-ID horses. Although the matrix of the pellets supplied minor amounts of NSC, there was no difference in the plasma glucose response between the horse groups, implying that the exacerbated insulin surge in ID horses occurred in response to dietary AA. The precise mechanisms for postprandial hyperinsulinemia in horses are not fully understood but may involve alterations in pancreatic sensitivity to nutrients and incretins or reduced insulin clearance rates (54). A similar response was observed in a previous study where ID horses had a 9-fold greater insulin response to a large protein meal (1.2 g CP/kg BW) compared to that of their non-ID counterparts (23). While we only provided a fifth (i.e., 0.25 g CP/kg BW) of the protein amount used in the previous study, we still observed significant elevation in plasma insulin concentrations in ID horses. Average peak plasma insulin concentrations for both protein sources were ~100 and 30 μIU/mL for the ID compared to the non-ID horses, respectively. Although a significantly greater plasma insulin response to the higher quality protein supplement was anticipated, hyperinsulinemia following consumption of the alfalfa pellet was unexpected. Peak concentrations were 95 μIU/mL in ID horses 1 h post feeding, levels that would commonly be considered as postprandial hyperinsulinemia. While alfalfa is sometimes favored for its lower NSC content in comparison to many grasses, these data indicate that protein levels should also be carefully evaluated when selecting forages for horses sensitive to ID.

The overall plasma amino acid response to both protein treatments was similar between horse groups, indicating digestion and absorption kinetics as well as amino acid removal from circulation (i.e., disposal or uptake into tissues) did not seem to be significantly altered by insulin status. ID horses did tend to have slightly higher peak leucine and non-EAA concentrations and AA clearance rates from circulation seemed to be somewhat delayed, which is in accordance to our previous study (23). There is still a paucity of data on postprandial AA responses in hyperinsulinemic individuals, but it is plausible that underlying insulin resistance (IR) could affect amino acid metabolism or uptake into tissues.

There were no differences in the degree of phosphorylation of mTOR and rpS6 between the ID and non-ID groups for either protein source, indicating that AA uptake by the muscle and subsequent mTOR pathway activation in response to a protein meal was not impaired in ID horses. These data are in accordance with previous work in horses using a glucocorticoid-induced model of IR where mTOR pathway activation in response to a protein meal was not different from healthy control horses despite clear evidence of IR (55). This study extends these findings in horses with naturally occurring ID. IR in humans and rodents has been associated with anabolic resistance and muscle wasting (56). However, in most instances of human diabetes, insufficient inhibition of protein breakdown by general absence of insulin is the primary cause for increased net loss of muscle protein (57, 58). In contrast, equine ID is rarely associated with insufficient insulin release but rather excessive postprandial hyperinsulinemia. While we did not assess peripheral tissue IR in this study, the same animals were used in an immediate follow-up project (59) where insulin sensitivity, as assessed by a combined glucose-insulin tolerance test, was shown to be reduced in this set of ID horses. A few other studies have illustrated alterations in GLUT-4 expression and GSK activation in muscle of IR horses, however, it should be noted that these signaling events occur upstream of mTOR and are under direct influence of insulin mediated signal transduction. Moreover, it is known that insulin facilitates, but is not required for AA-mediated mTOR pathway stimulation (12). Oral AA intake, and in particular leucine, can increase phosphorylation of mTOR and its downstream effectors 4E-BP-1 and S6K1 *via* Rheb-Ras proteins, and this response is maintained in skeletal muscle of diabetic rodents (47). Further research is needed to determine whether equine ID affects insulin signal transduction upstream of mTOR or prevents inhibition of proteolytic pathways which could potentially lead to muscle wasting over time. However, it can be concluded from the current data that ID does not prevent muscle mTOR and downstream rpS6 activation by dietary AA in horses.

Regardless of treatment, ID horses had overall higher levels of phosphorylated rpS6. A similar response was observed post feeding a protein meal in glucocorticoid-induced IR horses (55). Considering ID horses tended to have slightly higher peak plasma leucine concentrations, this may have provided stronger stimulation of S6K1 and subsequently rpS6 as it has been shown that leucine can also directly increase S6K phosphorylation downstream of mTOR (44). Alternatively, studies in rodents and humans have illustrated that S6K1, plays a role the development of cellular insulin resistance. Hyperactivation of S6K1 under nutrient abundancy has shown to initiate a negative feedback loop leading to decreased cellular insulin signal transduction (53). Although we did not measure S6K1 activity, it is the protein kinase directly responsible for rpS6 activation. It is therefore reasonable to assume elevated rpS6 phosphorylation levels could be reflective of a hyperactive S6K1 in ID horses.

In conclusion, the current study is the first in horses to show the time course of postprandial skeletal muscle mTOR pathway activation and compare the impact of dietary protein source on degree of activation of mTOR signaling proteins. We show that protein supplementation increases muscle mTOR pathway activation and that the maximal degree of phosphorylation of mTOR and rpS6 proteins occurs at ~90 min post-feeding with a subsequent dephosphorylation of these signaling proteins over a 5 h period. Additionally, we show that the consumption of a protein supplement containing a mixture of higher quality protein sources, tended to result in greater postprandial phosphorylation of mTOR and rpS6 proteins compared to equal amounts of crude protein from a forage-based protein source. Finally, we show that ID does not impair postprandial activation of mTOR and rpS6 proteins in horses following a protein-rich meal.

## Data availability statement

The raw data supporting the conclusions of this article will be made available by the authors, without undue reservation.

## Ethics statement

The animal study was reviewed and approved by University of Kentucky Institutional Animal Care and Use Committee.

## Author contributions

CL, KM, RC, DD, and KU: conception and design of research. CL, SS, and AB: performed experiment and lab analyses. CL, KM, and EV: analyzed data. CL, EV, KM, and KU: interpreted results of experiments. CL: prepared figures and drafted manuscript. CL, KM, EV, RC, and KU: edited and revised manuscript. CL, KM, EV, AB, SS, RC, DD, and KU: approved final version of manuscript. All authors contributed to the article and approved the submitted version.

## Funding

This project was supported by Versele-Laga (Deinze, Belgium) and the National Institute of Food and Agriculture, U.S. Department of Agriculture Hatch Program under KY0070109. Mention of trade name, proprietary product, or specified equipment does not constitute a guarantee or warranty by the University of Kentucky and does not imply approval to the exclusion of other products that may be available.

## Conflict of interest

DD was hired as a consultant by the funder of this project (Versele-Laga, Deinze, Belgium). He also receives a portion of the profits from Cavalor VitAmino. DD is employed by Equivado Consultancy B.V. The funding company did not participate in the analysis or the decision to publish. All authors declare that they had full autonomy and independency in the research and publishing of this work. The remaining authors declare that the research was conducted in the absence of any commercial or financial relationships that could be construed as a potential conflict of interest.

## Publisher's note

All claims expressed in this article are solely those of the authors and do not necessarily represent those of their affiliated organizations, or those of the publisher, the editors and the reviewers. Any product that may be evaluated in this article, or claim that may be made by its manufacturer, is not guaranteed or endorsed by the publisher.
