# Supplementary material for: Differential effect of two dietary protein sources on time course response of muscle anabolic signaling pathways in normal and insulin dysregulated horses
Source: Front Vet Sci. 2022 Aug 1;9:896220. doi: 10.3389/fvets.2022.896220 (PMC9376591; doi:10.3389/fvets.2022.896220)
Supplement: Supplementary file 1 [file Table_1.DOCX]

**Supplementary Table 1 Effects of protein source and insulin status on postprandial plasma EAA, leucine and non-EAA concentrations and relative abundance of phosphorylated rpS6 and mTOR proteins in gluteal muscle samples**

|  | **Alfalfa pellets** | | **Protein supplement** | | **P-values** | | | | | | |
| --- | --- | --- | --- | --- | --- | --- | --- | --- | --- | --- | --- |
|  | **Non-ID** | **ID** | **Non-ID** | **ID** | **Treat** | **INS status** | **Treat *INSstatus** | **Time** | **Treat *time** | **INSstatus *time** | **Treat*INS status *time** |
| **mTOR-P** (AU) |  |  |  |  | 0.48 | 0.87 | 0.58 | 0.0002 | 0.03 | 0.31 | 0.66 |
| 0 | 0.45 ± 0.15 | 0.52 ± 0.16 | 0.55 ± 0.16 | 0.57 ± 0.16 |  |  |  |  |  |  |  |
| 90 | 0.82 ± 0.19 | 0.82 ± 0.20 | 1.29 ± 0.29 | 1.07 ± 0.29 |  |  |  |  |  |  |  |
| 180 | 0.73 ± 0.18 | 0.64 ± 0.19 | 0.73 ± 0.21 | 0.72 ± 0.21 |  |  |  |  |  |  |  |
| 300 | 0.49 ± 0.18 | 0.77 ± 0.19 | 0.39 ± 0.16 | 0.46 ± 0.16 |  |  |  |  |  |  |  |
|  |  |  |  |  |  |  |  |  |  |  |  |
| **Rps6-P** (AU) |  |  |  |  | 0.08 | 0.007 | 0.84 | < 0.0001 | 0.10 | 0.72 | 0.35 |
| 0 | 0.12 ± 0.14 | 0.28 ± 0.14 | 0.12 ± 0.19 | 0.56 ± 0.19 |  |  |  |  |  |  |  |
| 90 | 0.66 ± 0.20 | 0.84 ± 0.22 | 0.95 ± 0.34 | 1.40 ± 0.34 |  |  |  |  |  |  |  |
| 180 | 0.40 ± 0.18 | 0.63 ± 0.20 | 0.71 ± 0.20 | 0.75 ± 0.20 |  |  |  |  |  |  |  |
| 300 | 0.14 ± 0.16 | 0.58 ± 0.17 | 0.12 ± 0.17 | 0.36 ± 0.17 |  |  |  |  |  |  |  |
|  |  |  |  |  |  |  |  |  |  |  |  |
| **EAA (**µmol/L) |  |  |  |  | 0.0004 | 0.99 | 1.0 | < 0.0001 | < 0.0001 | 0.64 | 0.99 |
| 0 | 730.2 ± 14.49 | 740.11 ± 16.72 | 731.86 ± 13.03 | 728.21 ± 13.08 |  |  |  |  |  |  |  |
| 15 | 776.51 ± 22.67 | 784.16 ± 26.17 | 754.33 ± 20.22 | 760.3 ± 20.25 |  |  |  |  |  |  |  |
| 30 | 789.25 ± 27.28 | 778.92 ± 31.5 | 802.97 ± 37.68 | 852.76 ± 37.7 |  |  |  |  |  |  |  |
| 60 | 841.01 ± 29.12 | 813.06 ± 33.62 | 962.64 ± 63.78 | 970.13 ± 63.79 |  |  |  |  |  |  |  |
| 90 | 853.34 ± 24.14 | 811.81 ± 27.87 | 1096.14 ± 48.94 | 1031.23 ± 48.95 |  |  |  |  |  |  |  |
| 120 | 817.13 ± 30.7 | 779.3 ± 37.52 | 1016.22 ± 47.87 | 948.1 ± 47.88 |  |  |  |  |  |  |  |
| 150 | 862.43 ± 35.37 | 835.32 ± 43.79 | 1016.61 ± 50.7 | 954.26 ± 50.72 |  |  |  |  |  |  |  |
| 180 | 849.74 ± 35.3 | 811.59 ± 44.02 | 1041.81 ± 53.91 | 963.94 ± 47.92 |  |  |  |  |  |  |  |
| 210 | 795 ± 26.45 | 833.44 ± 30.54 | 891.64 ± 41.28 | 882.48 ± 41.3 |  |  |  |  |  |  |  |
| 240 | 850.41 ± 41.69 | 884.28 ± 48.14 | 905.8 ± 54.86 | 910.49 ± 54.87 |  |  |  |  |  |  |  |
| 300 | 817.3 ± 33.59 | 845.67 ± 38.78 | 800.57 ± 44.21 | 851.08 ± 44.23 |  |  |  |  |  |  |  |
| 360 | 814.26 ± 21.48 | 856.71 ± 24.04 | 779.62 ± 32.8 | 861.09 ± 32.82 |  |  |  |  |  |  |  |
| 420 | 765.13 ± 28.38 | 774.12 ± 32.76 | 778.48 ± 31.76 | 835.66 ± 31.78 |  |  |  |  |  |  |  |
| 480 | 761.64 ± 32.75 | 778.72 ± 37.81 | 772.65 ± 28.71 | 797.73 ± 28.73 |  |  |  |  |  |  |  |
|  |  |  |  |  |  |  |  |  |  |  |  |
|  | **Suppl. Table 1 continued** | |  |  |  |  |  |  |  |  |  |
| **Leucine (**µmol/L) |  |  |  |  | 0.02 | 0.38 | 0.23 | < 0.0001 | < 0.0001 | 0.51 | 0.81 |
| 0 | 90.36 ± 3.16 | 92.87 ± 3.48 | 92.01 ± 2.58 | 90.22 ± 2.59 |  |  |  |  |  |  |  |
| 15 | 104.86 ± 4.73 | 101.87 ± 5.36 | 92.7 ± 5.46 | 93.29 ± 5.46 |  |  |  |  |  |  |  |
| 30 | 102.45 ± 5.9 | 94.16 ± 6.73 | 97.31 ± 6.71 | 108.86 ± 6.71 |  |  |  |  |  |  |  |
| 60 | 106.46 ± 6.98 | 96.97 ± 7.99 | 117.94 ± 10.53 | 122.05 ± 10.53 |  |  |  |  |  |  |  |
| 90 | 108.26 ± 4.66 | 91.22 ± 5.27 | 141.16 ± 7.31 | 128.47 ± 7.31 |  |  |  |  |  |  |  |
| 120 | 107.02 ± 7.28 | 91.81 ± 8.86 | 128.43 ± 8.6 | 119.46 ± 8.6 |  |  |  |  |  |  |  |
| 150 | 113.22 ± 7.05 | 99.61 ± 8.69 | 124.07 ± 9.14 | 115.33 ± 9.14 |  |  |  |  |  |  |  |
| 180 | 107.96 ± 5.78 | 96.19 ± 7.12 | 122.21 ± 8.44 | 116.64 ± 7.43 |  |  |  |  |  |  |  |
| 210 | 97.92 ± 4.96 | 99.02 ± 5.63 | 102.48 ± 7.34 | 104.48 ± 7.34 |  |  |  |  |  |  |  |
| 240 | 109.64 ± 7.43 | 107.55 ± 8.52 | 109.2 ± 8.58 | 113.47 ± 8.58 |  |  |  |  |  |  |  |
| 300 | 100.98 ± 7.11 | 102.89 ± 8.13 | 94.49 ± 7.7 | 101.77 ± 7.7 |  |  |  |  |  |  |  |
| 360 | 103.95 ± 4.61 | 105.52 ± 5.04 | 93.43 ± 5.8 | 104.74 ± 5.8 |  |  |  |  |  |  |  |
| 420 | 96.73 ± 4.51 | 88.02 ± 5.1 | 90.67 ± 5.08 | 98.88 ± 5.08 |  |  |  |  |  |  |  |
| 480 | 98.23 ± 3.81 | 89.27 ± 4.26 | 93.34 ± 4.88 | 93.61 ± 4.89 |  |  |  |  |  |  |  |
|  |  |  |  |  |  |  |  |  |  |  |  |
| **Non-EAA (**µmol/L) |  |  |  |  | 0.003 | 0.90 | 0.50 | < 0.0001 | 0.007 | 0.64 | 0.91 |
| 0 | 2337.08 ± 38.44 | 2374.93 ± 45.07 | 2327.08 ± 39.08 | 2348.52 ± 38.48 |  |  |  |  |  |  |  |
| 15 | 2388.82 ± 58.42 | 2432.49 ± 67.92 | 2337.03 ± 58.84 | 2382.47 ± 58.45 |  |  |  |  |  |  |  |
| 30 | 2451.33 ± 85.69 | 2440.22 ± 99.26 | 2430.24 ± 85.98 | 2545.64 ± 85.71 |  |  |  |  |  |  |  |
| 60 | 2609.79 ± 155.2 | 2510.17 ± 174.53 | 2683.35 ± 151.16 | 2877.62 ± 151 |  |  |  |  |  |  |  |
| 90 | 2606.99 ± 78.84 | 2475.96 ± 91.37 | 2936 ± 79.15 | 2916.39 ± 81.03 |  |  |  |  |  |  |  |
| 120 | 2544.69 ± 82.86 | 2441.86 ± 101.11 | 2876.23 ± 83.16 | 2792.11 ± 82.88 |  |  |  |  |  |  |  |
| 150 | 2553.14 ± 88.24 | 2491.35 ± 109.01 | 2874.9 ± 88.52 | 2780.87 ± 88.26 |  |  |  |  |  |  |  |
| 180 | 2647.78 ± 95.79 | 2472.88 ± 118.98 | 3016.07 ± 101.93 | 2852.12 ± 91.71 |  |  |  |  |  |  |  |
| 210 | 2526.59 ± 79.98 | 2593.34 ± 92.69 | 2797.52 ± 80.29 | 2708.71 ± 82.3 |  |  |  |  |  |  |  |
| 240 | 2514.75 ± 121.75 | 2656.35 ± 140.81 | 2776.7 ± 121.96 | 2734.84 ± 121.77 |  |  |  |  |  |  |  |
| 300 | 2542.39 ± 105.26 | 2562.09 ± 126.54 | 2548.74 ± 105.5 | 2624.83 ± 105.28 |  |  |  |  |  |  |  |
| 360 | 2544.15 ± 81.74 | 2583.5 ± 92.14 | 2479.7 ± 79.81 | 2744.08 ± 84.62 |  |  |  |  |  |  |  |
| 420 | 2453.44 ± 86.47 | 2379.98 ± 100.16 | 2424.55 ± 86.76 | 2661.95 ± 86.49 |  |  |  |  |  |  |  |
| 480 | 2370.02 ± 94.03 | 2292.02 ± 108.86 | 2410.17 ± 94.3 | 2543.11 ± 94.05 |  |  |  |  |  |  |  |

Abbreviations: mTOR-P: phosphorylated mechanistic target of rapamycin protein; rpS6-P: phosphorylated ribosomal S6-kinase protein; AU: arbitrary units; EAA: essential amino acids; INSstatus: effect of insulin status; Treat: effect of protein source; ID: insulin dysregulated. Essential AA: sum of all essential amino acids (i.e., histidine, threonine, valine, methionine, isoleucine, leucine, phenylalanine, tryptophan, lysine); non-essential AA: sum of all non-essential amino acids (i.e., aspartate, glutamate, serine, asparagine, glycine, glutamine, citrulline, alanine, arginine, proline, tyrosine, ornithine). Time 0-480 min: plasma and muscle samples taken before and at regular intervals post feeding the protein treatments. n = 8 ID and 8 non-ID horses. All data are lsmeans ± SE.
